# Unique tau‐ and synuclein‐dependent metabolic reprogramming in neurons distinct from normal aging

**DOI:** 10.1111/acel.14277

**Published:** 2024-08-13

**Authors:** Shweta Yadav, Aidan Graham, Farazdaq Al Hammood, Chris Garbark, Deepika Vasudevan, Udai Pandey, John M. Asara, Dhivyaa Rajasundaram, Andrey A. Parkhitko

**Affiliations:** ^1^ Aging Institute of UPMC and the University of Pittsburgh Pittsburgh Pennsylvania USA; ^2^ Department of Cell Biology University of Pittsburgh Pittsburgh Pennsylvania USA; ^3^ Department of Pediatrics, Children's Hospital of Pittsburgh University of Pittsburgh Pittsburgh Pennsylvania USA; ^4^ Division of Signal Transduction, Beth Israel Deaconess Medical Center, and Department of Medicine Harvard Medical School Boston Massachusetts USA

**Keywords:** AD, aging, *Drosophila*, GLS, glutaminase, metabolism, metabolomics, PD, PRAT/PPAT, two‐hit model

## Abstract

Neuronal cells are highly specialized cells and have a specific metabolic profile to support their function. It has been demonstrated that the metabolic profiles of different cells/tissues undergo significant reprogramming with advancing age, which has often been considered a contributing factor towards aging‐related diseases including Alzheimer's (AD) and Parkinson's (PD) diseases. However, it is unclear if the metabolic changes associated with normal aging predispose neurons to disease conditions or a distinct set of metabolic alterations happen in neurons in AD or PD which might contribute to disease pathologies. To decipher the changes in neuronal metabolism with age, in AD, or in PD, we performed high‐throughput steady‐state metabolite profiling on heads in wildtype *Drosophila* and in *Drosophila* models relevant to AD and PD. Intriguingly, we found that the spectrum of affected metabolic pathways is dramatically different between normal aging, Tau, or Synuclein overexpressing neurons. Genetic targeting of the purine and glutamate metabolism pathways, which were dysregulated in both old age and disease conditions partially rescued the neurodegenerative phenotype associated with the overexpression of wildtype and mutant tau. Our findings support a “two‐hit model” to explain the pathological manifestations associated with AD where both aging‐ and Tau/Synuclein‐ driven metabolic reprogramming events cooperate with each other, and targeting both could be a potent therapeutic strategy.

## Graphical abstract

We propose a “two‐hit model” to explain the pathological manifestations associated with AD/PD where both age‐ and Tau/Synuclein‐driven metabolic reprogramming events cooperate with each other and targeting both could be a potent therapeutic strategy.

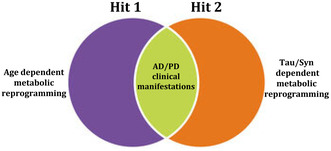

## 1 INTRODUCTION

Aging is one of the primary risk factors for many major human pathologies, including cancer, diabetes, cardiovascular disorders, and neurodegenerative diseases (Lopez‐Otin et al., 2013). Untargeted and targeted metabolomics analysis in worms (Fuchs et al., 2010), flies (Avanesov et al., 2014; Hoffman et al., 2014; Parkhitko et al., 2016), mice (Tomas‐Loba et al., 2013), and humans (Yu et al., 2012) have documented changes in metabolome during the aging process. We have previously shown that targeting these metabolic pathways that change with age can extend health‐ and lifespan (Parkhitko et al., 2020a). Despite these studies, not much is known about how metabolism is reprogrammed with age at the tissue‐specific level and whether such aging‐associated metabolic changes are similar or distinct to the metabolic changes associated with aging‐related diseases. The human brain is one of the most metabolically demanding organs and at resting state it accounts for nearly 20% of the body's total energy consumption (Magistretti & Allaman, 2015; Raichle & Gusnard, 2002). Neurons have high energy demands and concomitantly high metabolic rates which is crucial to support their function. Consequently, neurons are sensitive towards metabolic perturbations and several studies suggest aging‐dependent reprogramming of brain metabolism. Transcriptional analysis using RNA‐seq from the liver, kidney, and brain of 33 diverse species of mammals followed by gene set enrichment analysis revealed central energy metabolism as one of the most relevant biological pathways associated with life expectancy (Fushan et al., 2015). Increased lactate levels have been observed in cerebrospinal fluid in aging humans (Yesavage et al., 1982), and in brains from aged mice (Ross et al., 2010) and *Drosophila* (Long et al., 2020). Similarly, several studies have reported dysregulation of metabolic pathways in neurodegenerative diseases, such as a shift from glycolytic to oxidative metabolism in the parieto‐temporal brain regions of AD patients (Fukuyama et al., 1994).

*Drosophila* serves as a reliable model to discover metabolic changes associated with aging and neurodegeneration since most metabolic pathways and pathogenic mechanisms are conserved between flies and humans. Robust *Drosophila* models of multiple neurodegenerative diseases including AD, PD, and Huntington's disease have been established by targeted expression of neurodegeneration‐associated human proteins (Feany & Bender, 2000; Greeve et al., 2004; Jackson et al., 1998; Jackson et al., 2002; Nitta & Sugie, 2022; Rahul and Siddique, 2022; Warrick et al., 1998; Wittmann et al., 2001). Mutation in the gene coding for α‐synuclein (SNCA) was first reported in a familial case of PD (Polymeropoulos et al., 1997). Since then, multiple studies have shown that mutations or higher gene copies of SNCA are sufficient to recapitulate some aspects of PD manifestations in model organisms (Magalhaes & Lashuel, 2022). Notably, α‐synuclein aggregates are not unique to PD and have been found in the brains of patients suffering from a range of similar neurodegenerative diseases (collectively referred to as α‐synucleinopathies) including dementia with Lewy bodies (DLBs), specific cases of AD, and neurodegeneration with brain iron accumulation (NBIA) type I (Kahle, 2008). PD was first modeled in *Drosophila* by expressing human α‐Synuclein (Feany & Bender, 2000). The *Drosophila* transgenic model recapitulates the major hallmarks of PD such as progressive loss of dopaminergic neurons, locomotor deficits, and intra‐neuronal accumulation of inclusion bodies. Subsequently, the *Drosophila* model has also been used to study multiple aspects of PD including non‐motor deficits, such as perturbations in sleep behavior and olfactory defects (Suzuki et al., 2022).

Tauopathies refers to a group of neurodegenerative disorders characterized by deposits of the microtubule associated protein Tau in neurons or/and glial cells (Gotz et al., 2019) including AD and Frontotemporal Dementia and Parkinsonism linked to chromosome 17 (FTDP‐17). Tau is known to be hyperphosphorylated in AD and contributes to the formation of neurofibrillary tangles (Grundke‐Iqbal et al., 1986). A point mutation in Tau (Tau^V337M^) is associated with FTDP‐17 (D'Souza et al., 1999; Goedert & Spillantini, 2000; Wittmann et al., 2001). By expressing human wild type tau as well as disease variants of tau, Feany's group established a *Drosophila* tauopathy model (Wittmann et al., 2001). Similar to the PD model, the *Drosophila* tauopathy model recapitulates the major defects as observed in humans although the formation of neurofibrillary tangles was not detected.

Whether the aging‐related changes in neuronal metabolism is similar or distinct from changes seen in PD or AD has not been systematically explored. Here, we report our results from the analyses of high‐throughput steady‐state metabolite profiles from *Drosophila* head samples across different ages and compared them to those expressing Synuclein or Tau. We demonstrate that targeting metabolic pathways common to old age and disease condition rescued the neurodegenerative phenotype associated with AD. Based on our findings, we propose a “two‐hit model” through which aging‐ and Synuclein/Tau‐ driven metabolic reprogramming events cooperate ultimately resulting in the pathological manifestations associated with AD or PD. Targeting these metabolic deficits alone provides a partial rescue of the neuronal death phenotype in eyes and is likely to potentiate existing treatment regimes.

## 2 RESULTS

### 2.1 *Drosophila* neurons exhibit age‐dependent metabolic reprogramming

**FIGURE 1:**
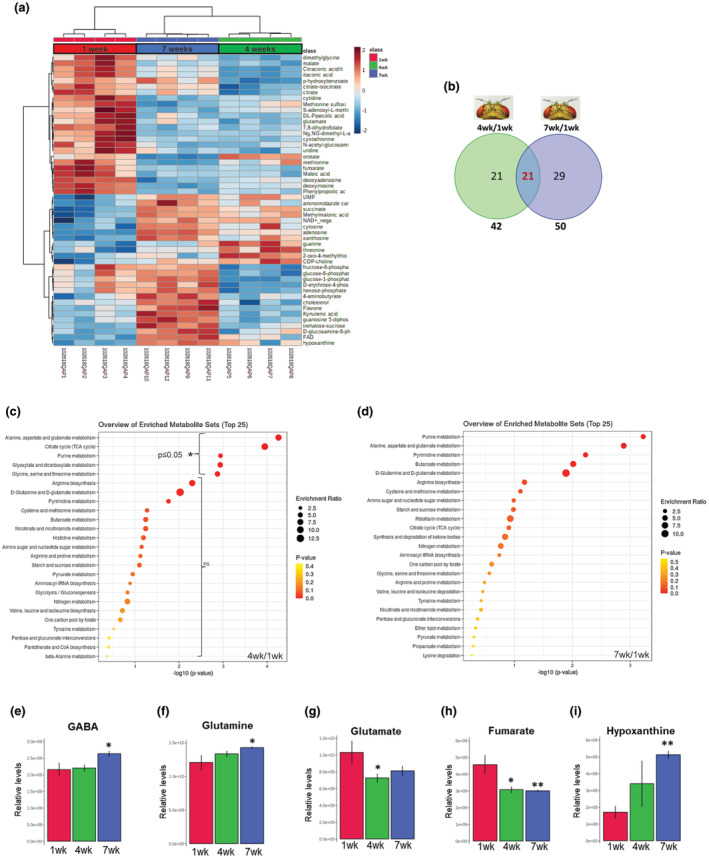
Metabolic reprogramming in *Drosophila* heads (a) Heat map showing the metabolites that were significantly changed in 1 week, 4 weeks and 7 weeks old heads of *OregonR* wildtype flies. Four replicates were run for each timepoint. The pseudocolor scaling of the standardized expression is from low (blue) to high (red) (b) Venn diagram showing the number of significantly altered metabolites at 4 weeks and 7 weeks compared to that at 1 week in fly head samples and the overlap between the two timepoints. (c, d) Metabolic Set Enrichment Analysis of the metabolites that changed significantly at 4 weeks and 7 weeks of age compared to 1 week. Color code reflects p‐value and the size of the corresponding circle reflects number of altered metabolites in the corresponding pathway. Pathways that were significantly altered are marked. (e–i) Relative levels of the specific metabolites in the heads at 1 week, 4 weeks and 7 weeks of age. Error bars represent standard deviation, (*p < 0.05; **p < 0.01; ***p < 0.001).

We previously performed high‐throughput steady‐state metabolite profiling of *Drosophila* whole‐body using targeted tandem mass spectrometry (LC–MS/MS) (Yuan et al., 2012) and discovered that the methionine and tyrosine metabolic pathways are reprogrammed with aging (Parkhitko et al., 2016; Parkhitko et al., 2020b). We and others also found that targeting methionine metabolism or the tyrosine degradation pathway either ubiquitously, or specifically in neuronal tissues, extends *Drosophila* health‐ and lifespan (Lee et al., 2014; Obata & Miura, 2015; Parkhitko et al., 2016; Parkhitko et al., 2019; Parkhitko et al., 2021). The analysis of the whole‐body (which includes head), where head constitutes only 10% of the total mass, may mask neuron‐specific aging‐dependent metabolic changes. Therefore, we performed high‐throughput steady‐state metabolite profiling on the *Drosophila* head, where the brain is estimated to be comprised of 90% neurons (Raji & Potter, 2021). We used the same targeted metabolomics platform covering 296 polar metabolites as before to compare our metabolomics datasets from the heads to the previously published datasets from the whole flies. We utilized a common *Drosophila* strain (*OregonR* [*OreR*]) aged at 1, 4, and 7 weeks corresponding to young‐, mid‐, and old‐age respectively. As we previously found that neuron‐specific manipulations of the methionine and tyrosine metabolism pathways affected lifespans only in female flies (Parkhitko et al., 2016; Parkhitko, Ramesh, et al., 2020), we used only female flies for metabolomics analysis in the heads. We observed striking progressive changes in distinct groups of metabolites across different ages (Figure 1a). When compared to young‐age (1 week), we found 42 and 50 unique metabolites to be significantly altered at mid‐age (4 weeks) and old age (7 weeks) respectively. We also found 21 metabolites that were common to both mid‐age and old‐age samples (Figure 1b; Table S1). Interestingly, like that observed in whole flies (Parkhitko et al., 2016), the changes in metabolite levels were bidirectional, as we detected both increase and decrease in metabolite levels with age. This suggests that the changes observed do not simply reflect a decrease in metabolic activity with age but rather provide evidence for aging‐dependent metabolic reprogramming at tissue level.

To understand which metabolic pathways were the most significantly affected with age, we applied Metabolite Set Enrichment Analysis (MSEA) to the set of differentially altered metabolites. After correcting for multiple hypothesis testing, we found that the purine metabolism pathway was significantly altered at both 4‐ and 7‐weeks (Figure 1c,d; Table S1). The 4‐week samples additionally showed distinct effects on the TCA cycle metabolites; alanine, aspartate and glutamate pathway; and glyoxylate metabolism pathway (Figure 1c; Table S1). Alterations in the levels of some of the key metabolites belonging to TCA cycle (fumarate), purine metabolism (hypoxanthine) and alanine, aspartate, and glutamate metabolism pathway [4‐aminobutyrate (GABA), glutamine and glutamate] are shown in (Figure 1e–i; Table S1). Altogether, we demonstrate that aging‐dependent metabolic reprogramming occurs in *Drosophila* heads (consisting mostly of neuronal tissue) and distinct metabolic pathways are specifically altered at mid‐age and old‐age neurons.

### 2.2 Neurons experience unique metabolic changes compared to the whole body

**FIGURE 2:**
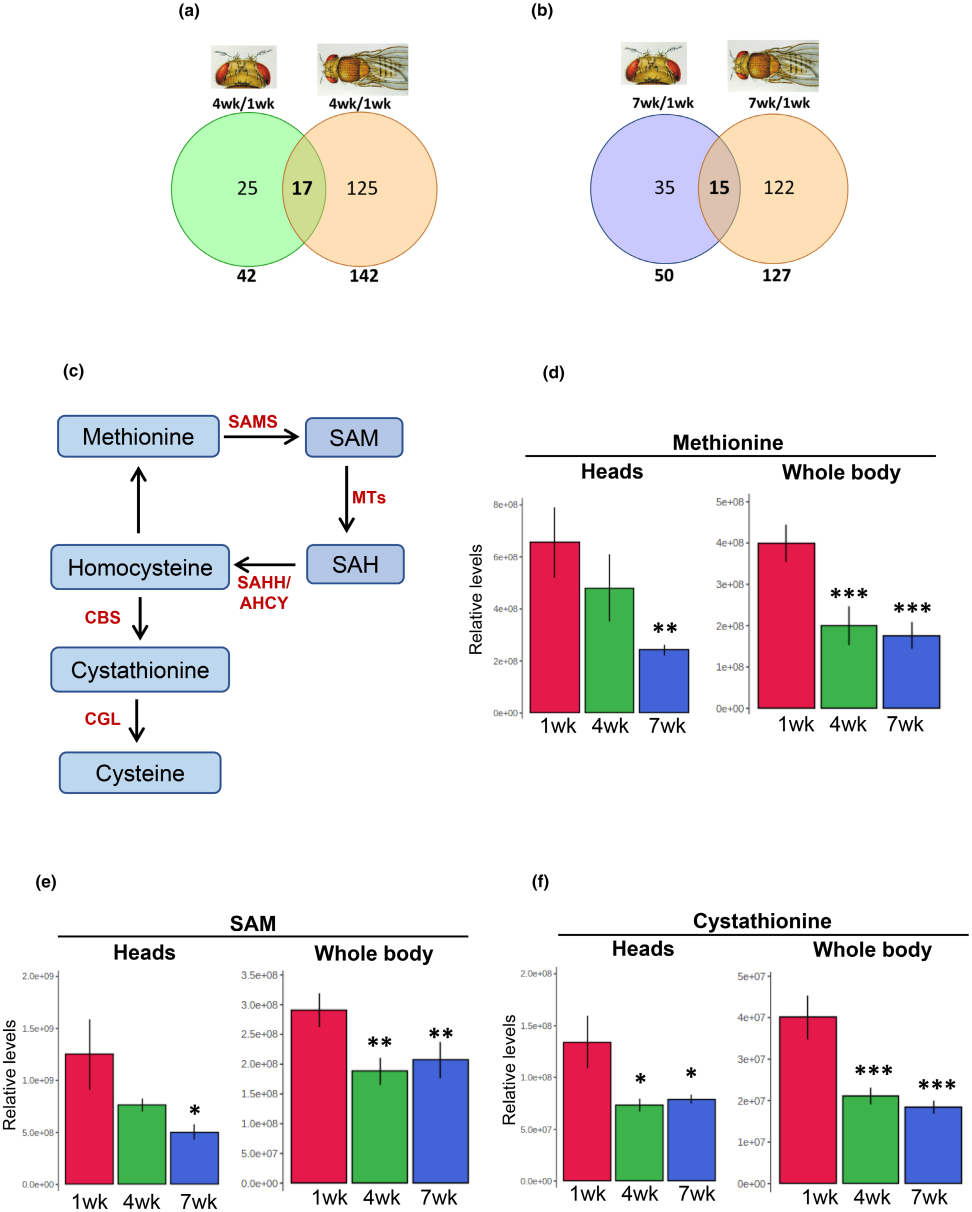
Comparison of the metabolic reprogramming in heads vs. whole body (a) Venn diagram showing the number of significantly altered metabolites in the head and whole‐body sample of 4 weeks old flies. (b) Venn diagram showing the number of significantly altered metabolites in the head and whole‐body sample of 7 weeks old flies. (c) Schematic diagram of methionine cycle showing key intermediates and the enzymes that catalyze these conversions. (d–f) Comparison of the levels of the specific intermediates in heads and whole‐body samples. Error bars represent standard deviation, (*p < 0.05; **p < 0.01; ***p < 0.001).

We next sought to determine which of the observed metabolic changes were specific to the *Drosophila* head in comparison to the whole body. A previous study used the transcriptomics approach to compare aging‐driven transcriptional responses in *Drosophila* head, thorax, or whole body (Girardot et al., 2006). They reported a significant overlap between the number of differentially expressed genes with age in the whole body and in different tissues. To access the differences between the age‐dependent metabolic changes in neurons vs. the whole body, we reanalyzed our previously published high‐throughput steady‐state metabolite profiling dataset on whole flies of the same *Drosophila* strain, sex, and age as we used for the head samples (Parkhitko et al., 2016). We identified 142 and 127 metabolites were significantly changed at 4‐weeks and 7‐weeks compared to that at 1‐week in whole flies (Figure 2a,b). Interestingly, the total number of significantly altered metabolites in the whole body is significantly higher compared to that in heads. This is consistent with a comparable microarray analysis between whole animals and heads, which found a higher number of significantly altered transcript levels in whole flies compared to heads (Girardot et al., 2006). Similar to this transcriptional study, we found both specific and common metabolic alterations across ages in whole animals and just heads (Figure 2a,b).

We have previously demonstrated that the steady state levels of multiple intermediates in the methionine metabolism pathway (Parkhitko et al., 2016) and methionine metabolic flux (Parkhitko et al., 2021) are altered during the aging process. We also demonstrated that these metabolic pathways contribute towards aging and targeting these pathways extends lifespan and healthspan (Parkhitko et al., 2016; Parkhitko et al., 2021). We looked at the levels of the key intermediates of methionine metabolism pathway in the head‐specific metabolomic profile to check if similar alterations in the methionine metabolism pathway were observed. Interestingly, we found that the levels of key intermediates in the methionine metabolism pathway namely methionine, S‐Adenosyl methionine (SAM), and cystathionine (Figure 2c) were reduced both in whole body as well as in heads of aged flies (Figure 2d–f). Altogether, we demonstrate that age‐dependent metabolic reprogramming in *Drosophila* heads shares some common metabolic pathways with that of whole‐body age‐dependent metabolic reprogramming, such as methionine metabolism. However, some of the age‐dependent metabolic changes are unique to heads.

### 2.3 Synuclein‐drives distinct metabolic reprogramming in *Drosophila* neurons

**FIGURE 3:**
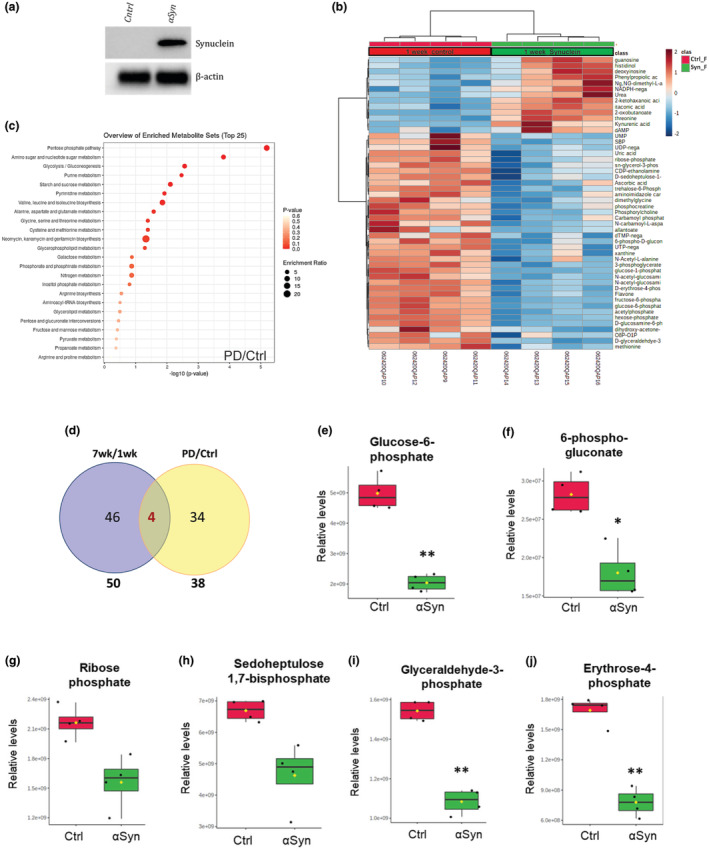
Unique metabolic signature associated with Synuclein expressing flies (a) Immunoblot showing expression of α‐synuclein in the heads of female flies. Ctrl refers to the genetic control line (b) Heat‐map depicting levels of altered metabolites in 1‐week old control flies and flies expressing Synuclein. The pseudocolor scaling of the standardized expression is from low (blue) to high (red). (c) Metabolic Set Enrichment Analysis of the metabolites that changed significantly in the heads of 1‐week old synuclein expressing flies. Color code reflects p‐value and the size of the corresponding circle reflects number of altered metabolites in the corresponding pathway. Pathways that were significantly altered are marked. (d) Venn diagram showing the number of significantly altered metabolites detected in synuclein expressing fly heads at 1 week compared to that in wildtype fly heads at 7 weeks and the overlap between the two dataset. (e–j) Box plots showing the relative levels of the mentioned metabolites in the heads of 1 week old Synuclein flies. Error bars represent standard deviation, (*p < 0.05; **p < 0.01; ***p < 0.001).

We next asked if the metabolic reprogramming associated with normal aging neurons is similar to the metabolic reprogramming associated with neurodegenerative diseases. We used a *Drosophila* α‐synucleinopathy model that is characterized by robust neurodegeneration, early‐onset locomotor deficits, and abundant α‐synuclein aggregation (Ordonez et al., 2018). We confirmed expression of α‐synuclein in the heads by immunoblotting (Figure 3a). To investigate the impact of α‐synuclein expression on the metabolism of neuronal cells, we performed the same high‐throughput steady‐state metabolite profiling on heads from either control flies or flies expressing wild‐type human α‐synuclein using synaptobrevin‐QF2 system (SybQF2) driver (Ordonez et al., 2018) at 1‐week of age (Figure 3b). We specifically used 1‐week old flies (young‐age) to test whether expression of synuclein could induce metabolic reprogramming similar to that associated with old age. Moreover, at this age these flies are known to exhibit the locomotor deficits and neuronal degeneration phenotypes which recapitulate some of the PD manifestations in humans (Ordonez et al., 2018). We detected 38 significantly altered metabolites in flies expressing synuclein (Figure 3d; Table S2). Based on the MSEA, the most significantly affected metabolic pathway with six significantly altered metabolites (Glucose‐6‐phosphate, Ribose‐phosphate, D‐sedoheptulose‐1‐7‐bisphosphate, D‐glyceraldehdye‐3‐phosphate, 6‐phospho‐D‐gluconate, and D‐erythrose‐4‐phosphate) was the pentose phosphate pathway (Figure 3c,e–j; Table S2). Alteration in the pentose phosphate pathway has also been reported in other models of PD as well as in PD patients (Chen et al., 2015; Poliquin et al., 2013; Tang, 2019; Tu et al., 2019). To examine whether the metabolic reprogramming seen in the PD model was similar to that observed with aging, we compared the significantly altered metabolites at 4‐ and 7‐week head samples with the significantly altered metabolites in the PD model. Surprisingly, only four significantly altered metabolites overlapped between 7‐week old flies and PD flies (guanosine, kynurenic acid, methionine, and N‐acetyl‐D‐glucosamine 6‐phosphate) and six significantly altered metabolites overlapped between 4‐week old flies and PD flies (D‐erythrose‐4‐phosphate, fructose‐6‐phosphate, glucose‐1‐phosphate, N‐acetyl‐glucosamine, threonine, and xanthine) (Figure S1a). Interestingly, one of these commonly altered metabolites is methionine (Figure S1b). Alterations in the levels of some of the key metabolites are shown in (Figure 3e–j). Altogether, we demonstrate that Synuclein expression in neurons drives metabolic reprogramming that differs from age‐dependent metabolic reprogramming.

### 2.4 Tau‐driven metabolic reprogramming in *Drosophila* neurons

**FIGURE 4:**
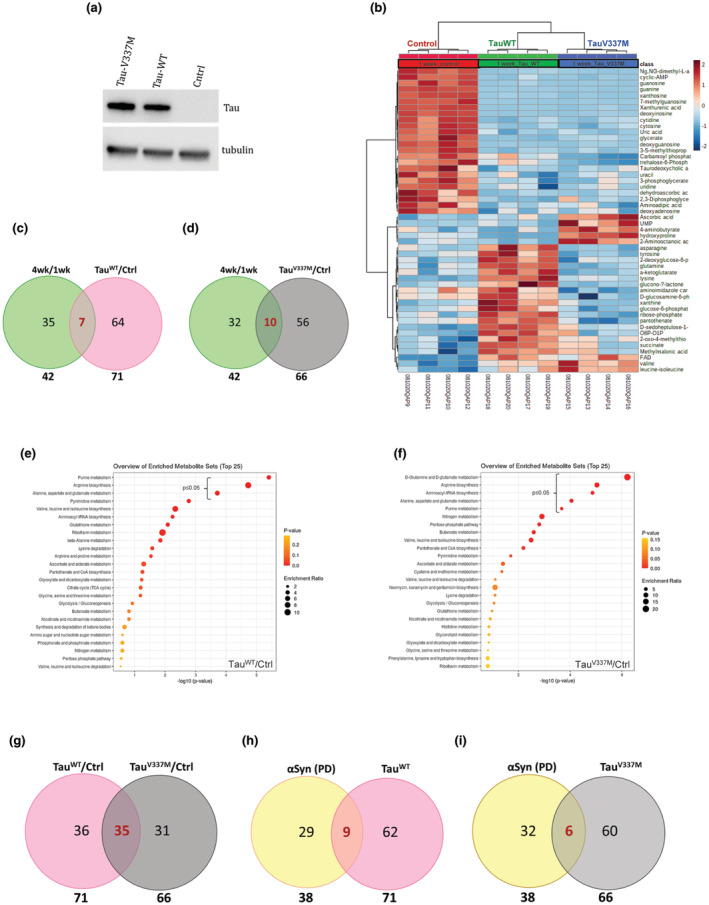
Unique metabolic signature associated with flies expressing wildtype and mutant Tau (a) Immunoblot image showing expression of Tau protein in the head extract of female flies expressing Tau‐WT and the Tau‐V337M. Ctrl refers to the genetic control line. (b) Heat‐map depicting levels of altered metabolites in 1‐week old control flies and flies expressing wildtype Tau (Tau‐WT) or mutant Tau (Tau‐V337M) using ElavGAL4 driver. The pseudocolor scaling of the standardized expression is from low (blue) to high (red). (c, d) Venn diagram showing the number of significantly altered metabolites detected in Tau‐WT and Tau‐V337M expressing fly heads at 1 week compared to that in wildtype fly heads at 4 weeks and the overlap between the respective datasets. (e, f) Metabolic Set Enrichment Analysis of the metabolites that changed significantly in the heads of 1‐week old flies expressing Tau‐WT and Tau‐V337M respectively. Color code reflects *p*‐value and the size of the corresponding circle reflects number of altered metabolites in the corresponding pathway. Pathways that were significantly altered are marked. (g) Venn diagram showing the extent of overlap between the numbers of significantly altered metabolites detected in fly heads expressing Tau‐WT and Tau‐V337M. (h) Venn diagram showing the extent of overlap between the numbers of significantly altered metabolites detected in fly heads expressing synuclein and Tau‐WT. (i) Venn diagram showing the extent of overlap between the numbers of significantly altered metabolites detected in fly heads expressing synuclein and Tau‐V337M.

We next tested a different model of neurodegeneration caused by the expression of Tau. It has been previously demonstrated that expression of human Tau (and especially mutant Tau) results in neurodegeneration in the *Drosophila* brain and is reflected by the appearance of vacuoles in the brain cortex (Wittmann et al., 2001). To investigate the impact of expression of either wild‐type Tau (Tau‐WT) or a specific mutant of Tau namely V337M (Tau‐V337M) on the metabolism in neuronal cells, we used flies expressing either of these proteins using neuronal promoter ElavGal4. We confirmed the expression of proteins in heads (Figure 4a) and performed the same high‐throughput steady‐state metabolite profiling on heads from either control flies or flies expressing Tau‐WT or Tau‐V337M using the pan‐neuronal Elav‐GAL4 driver at 1 week of age (Figure 4b). For our study, we chose to use the previously well characterized pan‐neuronal model of AD (Elav‐GAL4 driven human Tau) (Wittmann et al., 2001). We detected 71 significantly altered metabolites in the heads of flies expressing Tau‐WT (Figure 4c) and 66 significantly altered metabolites in the heads of flies expressing Tau‐V337M (Figure 4d). We found a substantial overlap between the two data sets (35 commonly altered metabolites between flies expressing Tau‐WT and Tau‐V337M) (Figure 4g; Table S3). We further performed MSEA on both sets of significantly altered metabolites and found three metabolic pathways including purine metabolism and arginine biosynthesis were significantly affected metabolic pathways in both data sets (Figure 4e,f; Table S3). This is in accordance with the previous reports where arginine metabolism has been shown to be involved in AD pathogenesis in humans. The levels of L‐arginine and its downstream metabolites have been reported to be altered in different regions of AD brains (Liu et al., 2014) and altered plasma arginine metabolome is known to precede behavioral changes in the APPswe/PS1ΔE9 mouse model of AD (Bergin et al., 2018). Besides arginine metabolism, the MSEA of flies expressing Tau‐WT as well as Tau‐V337M showed purine metabolism to be one of the significantly altered metabolic pathways (Figure 4e). The expression levels of genes coding for enzymes of purine metabolism pathway as well as the levels of metabolic intermediates have been reported to be altered in the brains of AD patients compared to controls (Ansoleaga et al., 2015). We further compared the overlap between significantly altered metabolites in the heads of flies expressing either Tau‐WT or Tau‐V337M to the significantly altered metabolites in the mid‐age and old‐age heads (Figure S1 c,d). Similar to the PD model, only 10 metabolites overlapped between Tau‐ and old‐age heads. Furthermore, we checked for shared alterations across PD and AD models. Interestingly there were only nine common metabolites between flies expressing Tau‐WT or Synuclein, and six common metabolites between flies expressing Tau‐V337M and Synuclein that were significantly altered (Figure 4h,i). Altogether, we demonstrate that Tau expression uniquely reprograms metabolism in *Drosophila* heads and some of these changes are conserved in the brains of human AD patients. Although both PD and AD share common neuronal manifestations like neuronal death, the metabolic reprogramming is unique to each model.

### 2.5 Integration of metabolomics data in *Drosophila* aged, Synuclein‐, or Tau expressing heads

**FIGURE 5:**
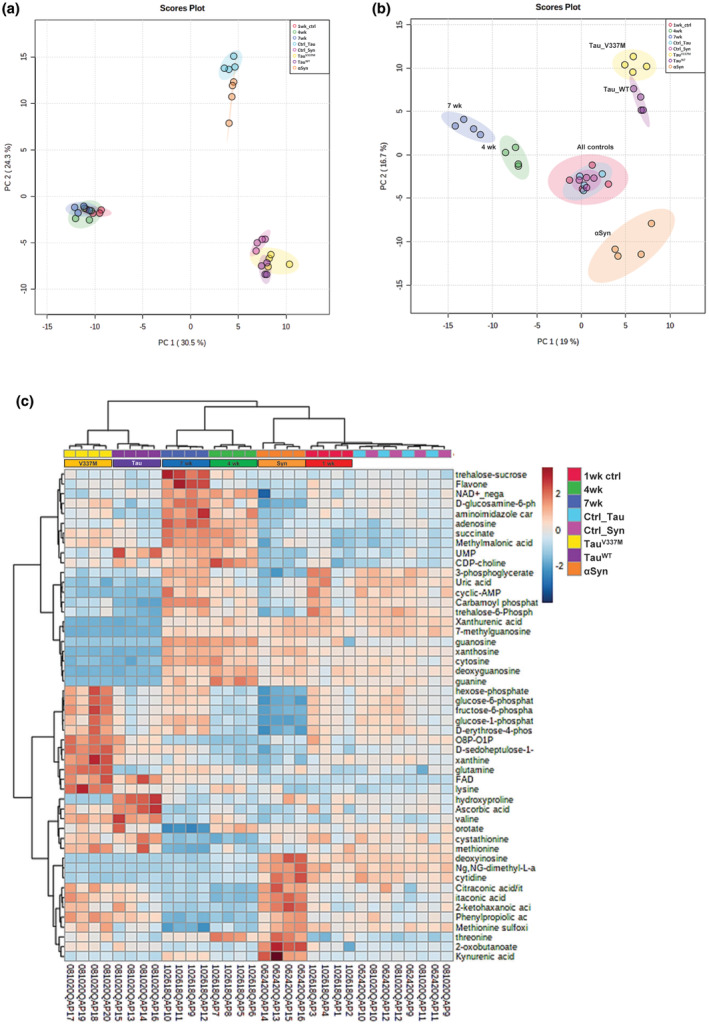
Comparison of the metabolic reprogramming with age and in the context of neurodegenerative models (a) Principal component analysis (PCA) of synuclein expressing heads, wildtype tau or mutant tau expressing heads, or from the aged heads. (b) PCA of synuclein expressing heads, wildtype tau or mutant tau expressing heads, or from the aged heads. All samples were normalized relative to the average of controls. The ellipses correspond to 95% confidence intervals. (c) Heat map showing the set of altered metabolites across different age groups and the neurodegenerative models.

To integrate all three datasets (from *Drosophila* aged, Synuclein‐, or Tau expressing heads), we analyzed all the datasets together using a Principal component analysis (PCA). As expected, a PCA of the measured metabolites clearly grouped all samples analyzed at the same time independent of the age/genotype of the flies (Figure 5a). To perform the comparisons between all the samples, we first normalized all the data relative to the control flies in each experiment and instead of comparing the absolute levels of the metabolites (areas under the peaks), we compared the relative changes to the average of controls. PCA of the normalized metabolites clearly grouped all control samples together (Figure 5b). The data from 4‐weeks and 7‐weeks old heads clustered closely, as did the data from animals expressing wildtype tau and mutant tau. In contrast, the data from synuclein, wildtype tau, mutant tau expressing heads, and the aged heads were far separated into different regions of the PCA plot (Figure 5b). This is further corroborated by unsupervised clustering of the metabolites, which places the aforementioned groups in different arms of the dendrogram (Figure 5c). These analyses support our conclusion that the spectrum of affected metabolites is dramatically different between normal aging, Synuclein, or Tau expression. In summary, unbiased integrative analysis of metabolic alterations suggests uniquely reprogramed metabolism in *Drosophila* aged, Synuclein, or Tau expressing heads.

### 2.6 Analysis of the enriched metabolic pathways in the human AD, PD, and AD/PD samples

**FIGURE 6:**
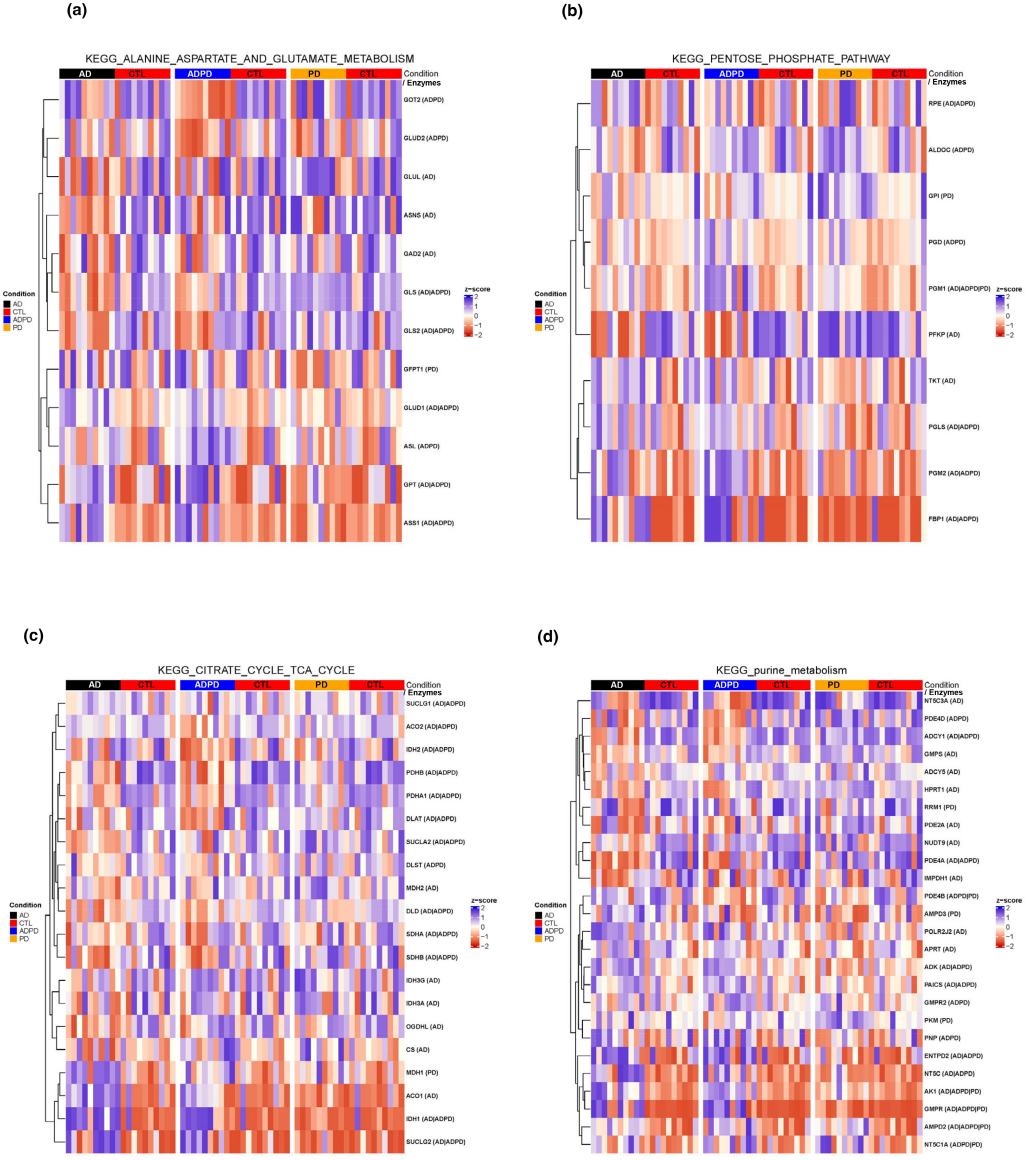
Proteomics analysis of metabolic enzyme levels in human brain samples from neurodegenerative disease patients (a–d) Heat maps showing the relative protein levels of metabolic enzymes belonging to mentioned metabolic pathways as measured in human brain samples. The significantly altered metabolites are marked for each group: (AD)—significantly altered in AD patients, (PD)—significantly altered in (PD) patients, (ADPD)—significantly altered in ADPD patients.

We next explored whether metabolic pathways reprogrammed in *Drosophila* heads are also altered in human brain samples of AD or PD. To do so, we relied on a previously published unbiased quantitative proteomic analysis of postmortem human brain tissues from four different groups defined as controls, AD, PD, and comorbid AD/PD cases (Ping et al., 2018). Predictably, this data set did not contain young brain samples. Moreover, the age‐dependent alterations would be mixed with the disease‐specific changes. To determine if our results on changes of metabolic pathways in *Drosophila* heads at the metabolome level were similar to those changes of metabolic pathways at the proteome level in human brains, we first extracted (from KEGG) all the enzymes attributed to the metabolic pathways that were significantly enriched based on our MSEA (Figure 1c,d; 3c and 4e,f). We chose four metabolic pathways namely the TCA cycle pathway, the purine metabolism pathway, the pentose phosphate pathway, and the alanine, aspartate and glutamate pathway for the comparison of the levels of various enzymes across control, AD, PD, and ADPD samples. We found that the levels of multiple enzymes from these pathways were significantly different in the human brain samples of AD, PD, or ADPD with the most enzymes attributed to ADPD samples (Figure 6a–d). It should be noted that in many cases the activity of enzymes is regulated via posttranslational modifications and the unchanged levels of enzymes do not necessarily mean that the activity of the specific metabolic pathway is unchanged. Similarly, changes in the levels of enzymes might not reflect the direction of the reprogrammed metabolic pathway. In summary, we found that the levels of multiple enzymes are altered in the brain samples of patients with AD, PD, and ADPD corroborating the changes observed in the respective metabolic pathways in the *Drosophila* heads.

### 2.7 Targeting tau‐associated metabolic reprogramming rescues the rough eye phenotype (REP) associated with the eye specific model of AD and FTDP

**FIGURE 7:**
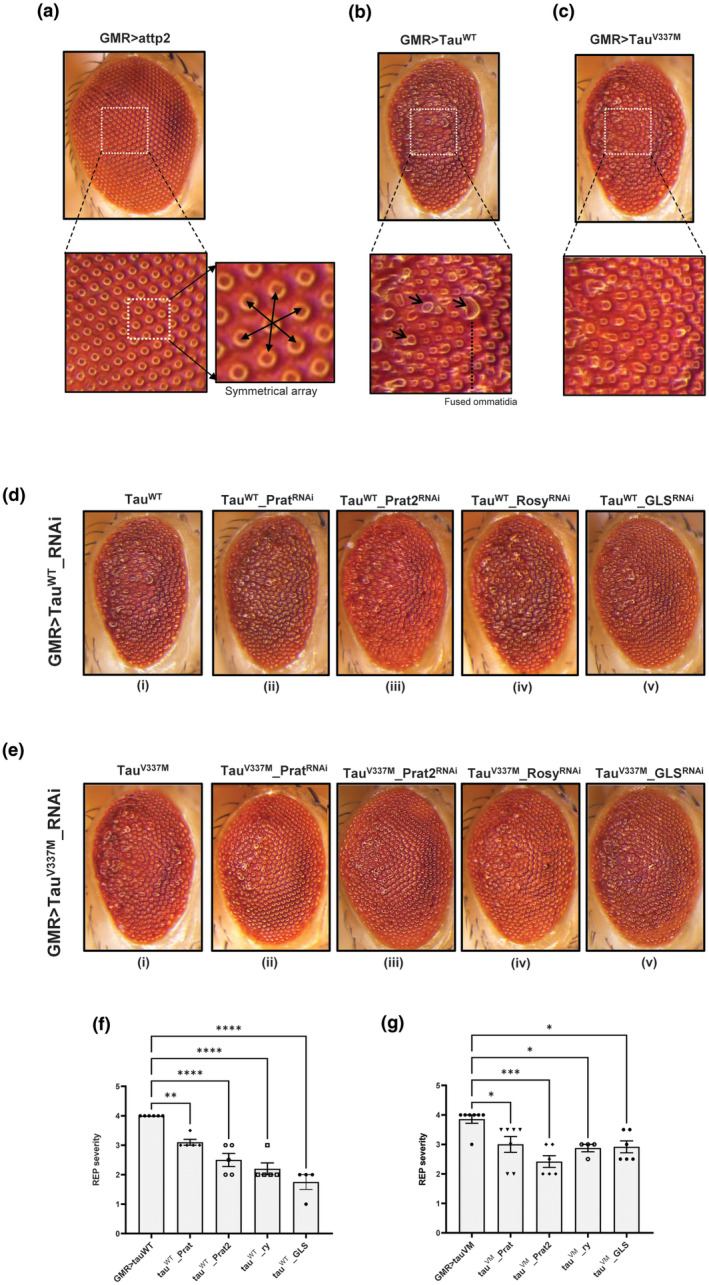
Perturbing purine and glutamate metabolism rescues the neurodegenerative phenotype in AD and FTDP models of *Drosophila*. Representative bright field images of *Drosophila* compound eye in (a) wildtype control flies (GMR > attp2) and in flies expressing Tau‐WT (b) or Tau‐V337M (c) using GMR‐GAL4. The inset shows a zoomed‐in view of the eye showing the arrangement of individual ommatidia and the ommatidial array. (d, e) Representative images of compound eye in flies of mentioned genotypes. (i–v) Five different enzymes were knocked down using RNAi lines. (f, g) Quantification of the rough eye phenotype (REP) in the mentioned genotypes. One way ANOVA was performed considering GMR_tau_WT or GMR_tau_VM as control.

Based on our metabolomics results and MSEA analysis, we found that purine metabolism was commonly affected in old age as well as in AD and FTD. Similarly, Ala/Asp/Glu metabolism was commonly affected at a young age and in AD/FTD. To test whether these metabolic changes are correlative or causal to the neuropathological manifestations associated with expression of wildtype tau and mutant tau, we manipulated purine metabolism and glutamate metabolism in flies expressing Tau‐WT or mutant tau (Tau‐V337M). To perturb glutamate metabolism, we used RNAi against glutaminase (GLS), the enzyme which converts glutamine to glutamate. To perturb the purine metabolism pathway, we knocked down the rate‐limiting enzyme of de novo purine biosynthesis pathway, namely phosphoribosylamidotransferase [orthologous to human phosphoribosyl pyrophosphate amidotransferase (PPAT)] which is encoded by two duplicate genes in *Drosophila*, *Prat*, and *Prat2*; and the key enzyme in purine degradation pathway, xanthine dehydrogenase (encoded by gene *rosy*). The expression of both human wildtype tau (Tau‐WT) as well as the mutant tau (Tau‐V337M) in *Drosophila* eyes using glass multiple reporter (GMR) driver results in the REP characterized by the reduced eye size, loss of curvature, disorganized ommatidial array, and presence of fused ommatidia (Figure 7a–c). To test the impact of perturbing purine metabolism on the REP associated with Tau‐WT or Tau‐V337M, we downregulated Prat and Prat2 using GMR‐GAL4. As shown in (Figure 7d‐ii), knockdown of *Prat* modified the REP in flies expressing Tau‐WT by partially restoring the curvature and reduced the number of fused ommatidia. Interestingly it rescued both the curvature as well as ommatidial array in flies expressing Tau‐V337M, besides reducing the number of fused ommatidia (Figure 7e‐ii). Knockdown of gene Prat2 modified the REP in Tau‐WT as well as in Tau‐V337M (Figure 7d‐iii and 7e‐iii). We quantified the level of knockdown of Prat and Prat2 (Figure S1f). Similarly, knockdown of the gene *rosy* modified Tau‐WT phenotype slightly but it significantly rescued the rough eye associated with Tau‐V337M (Figure 7d‐iv and 7e‐iv). Overall the results show that modulating purine anabolism or catabolism rescues the REP associated with tau expression, but the differential rescue observed across Tau‐WT and Tau‐V337M might be due to the differential metabolic dysregulations associated with these tauopathies. This also corroborates our initial finding where we found that Tau‐WT and Tau‐V337M have unique and distinct metabolic signatures.

Next, to test the impact of perturbing glutamate metabolism we knocked down the gene coding for GLS enzyme in flies expressing Tau‐WT and Tau‐V337M using a previously tested RNAi line. As shown in (Figure 7d–v and 7e–v) knocking down GLS significantly rescued the REP in both tauopathy models. In all cases refered above, we quantified the rough eye phenotype as shown in (Figure 7e and 7f). Altogether we show that purine and glutamate metabolism pathways are significantly affected in old age as well as in the AD/FTDP model of flies. Modulating both pathways rescued the neurodegenerative phenotype associated with AD and FTDP models which supports our two‐hit model where the absence of either aging‐induced metabolic dysfunction or Syn/Tau‐induced metabolic deficits would likely alleviate the disease‐associated pathological manifestations.

## 3 DISCUSSION

All organisms undergo a multitude of changes at cellular, tissue, and systemic level with increasing age, most of which are detrimental at the structural and/or functional level. Disruption of the metabolic homeostasis is one of the major hallmarks of aging and has been implicated as a contributing factor for several neurodegenerative diseases like AD, PD, Huntington's disease, and multiple sclerosis (Hou et al., 2019; Procaccini et al., 2016). The human brain has the highest energy demand compared with those of other organs in the body, and it has been known to change with age (Kuzawa et al., 2014). In our previous studies, we had performed the metabolomics on the whole‐body (Parkhitko et al., 2016). As the ‘head’ mass constitutes only 10% of the whole‐body mass, it does not reflect head/neuronal‐specific alterations. Hence, we performed similar metabolomics analysis for ‘heads’ alone. To decipher the changes in the neuronal metabolism with increasing age, we systematically performed targeted metabolomics on young‐, mid‐, and old‐age fly heads. We were interested in how neuronal metabolism reprograms with age, but we could not go for sorting of neurons as it would dramatically affect neuronal metabolome and is technically unfeasible. Since, *Drosophila* brain is estimated to be comprised of 90% neurons (Raji & Potter, 2021), they are considered to be a good proxy for reflecting neuronal‐specific changes. Comparative analysis of the data revealed various alterations in neuronal metabolism. Our data corroborates some of similar findings performed on other model organisms, for example, alterations in the levels of TCA cycle intermediates such as fumarate have been reported in aged mice (Dong & Brewer, 2019) and aged rats (Villa et al., 2009). Similarly, age‐dependent alterations in the levels of intermediates in the purine metabolism pathway (e.g., hypoxanthine) have also been reported in human cerebrospinal fluid (Peters et al., 2021) and in the aged mouse brain (Ivanisevic et al., 2016). Furthermore, the age‐dependent alterations of intermediates in the alanine, aspartate, and glutamate metabolism pathway have been observed in the human brain (Kaiser et al., 2005; Roalf et al., 2020). All of these examples reflect that the metabolic alterations observed in flies are conserved when compared to mice or humans.

By comparing metabolic changes in the heads of wild type flies during aging, as well as in flies expressing either Synuclein or Tau, we identified metabolic reprogramming signatures unique for each model of neurodegeneration. In our study, we used previously characterized *Drosophila* models relevant to PD and AD that exhibit manifestations similar to humans. As a result, we had to use genetic drivers that were originally described to create these models (for modeling PD, we used nSybQF2, and for modeling AD and FTD, we used Elav‐Gal4). We did not directly compare the data between different models; instead, we only compared the significantly altered metabolites/metabolic pathways across different time points in the respective models. Further, the use of neuronal‐specific drivers for the expression of Tau and synuclein ensures that the observed significantly altered metabolites likely represent neuronal‐specific reprogramming.

Some of the alterations of the metabolic pathways that we found to be enriched in the disease condition have also been reported in other models of aging/neurodegeneration reflecting that they are conserved across species, and this is further supported by our analysis of the proteomics data from the brains of AD, PD, and ADPD patients. Furthermore, we demonstrate that the observed alterations of the metabolic pathways are not just correlative. We found that targeting the rate‐limiting enzymes in the glutamate metabolism and purine metabolism pathway rescues the neurodegeneration in the eye model relevant to AD and FTDP.

### 3.1 Metabolic reprogramming: Steady state versus flux

Through our metabolomics analysis, we identified sets of metabolites belonging to specific metabolic pathways that were significantly altered at old age and in specific disease conditions. It should be noted that although alterations in the levels of particular metabolites may suggest altered pathway activity, increased levels of a specific metabolite can be attributed to either an increase or decrease in flux through this metabolic pathway. In addition to this, functional analysis should be performed to test whether these changes are causative or simply correlative. For example, we have previously demonstrated that steady state levels of methionine and other intermediates in the methionine metabolism pathway change with age; we further applied ^13^C5‐Methionine labeling to *Drosophila* and demonstrated significant changes in the activity of different branches of the methionine metabolism pathway in aging flies; and we found that altering methionine metabolism extends health‐ and lifespan. Similar analysis should be systematically performed in the future on the metabolic pathways that we found to be affected by aging or neurodegeneration in *Drosophila* heads. For this work, we selected several rate‐limiting enzymes in glutamate metabolism and purine metabolism pathways and demonstrated that their inhibition rescued the neurodegeneration phenotype in the eye model relevant to AD and FTDP.

### 3.2 Purine and glutamate metabolism

We used a targeted metabolomics approach and our findings are in agreement with previous reports from other labs. In a recent study focused on delineating the metabolic reprogramming that takes place during aging in *Drosophila*, Wang et al reported an increase in purine metabolism with advancing age which was supported by higher transcript levels of the key enzymes of purine metabolism (Wang et al., 2022). Using global isotope tracing metabolomics, they compared the tissue‐specific metabolic activities associated with heads and muscles in *Drosophila* and showed that purine metabolism was predominantly higher in heads. Our metabolic data from heads also reflected purine metabolism to be highly affected at old age.

GLS converts glutamine to glutamate and ammonia. Immunostaining‐based studies have shown positive correlation between the presence of neurofibrillary tangles in neurons in AD patient samples and GLS staining (Kowall & Beal, 1991). Fuchsberger et al showed that AD patient‐derived brain samples have elevated levels of GLS (Burbaeva et al., 2005). In our study we show that reducing GLS activity using RNAi rescues neurodegeneration in a *Drosophila* model of AD. This is in accordance with another study which showed that inhibition of GLS protected Aβ treated neurons from glutamate excitotoxicity and apoptosis (Fuchsberger et al., 2016). In humans, beneficial effects have also been demonstrated upon the inhibition of GLS in the glial system (Hollinger et al., 2020). Glutamate is an excitatory neurotransmitter and maintaining glutamate homeostasis in at synaptic sites is critically important for the proper brain function.

### 3.3 Two‐hit model

Since neuronal dysfunction is a fundamental part of aging, we expected to have similar metabolic alterations in neuronal populations associated with old age and in the context of neurodegenerative diseases. Our initial expectation was that we would find multiple common significantly altered metabolites between normal aging and different models of neurodegeneration reflecting a common signature of dying/dysfunctional neurons. However, the PCA analysis and unsupervised clustering clearly separated the groups of aged, Tau‐, or synuclein expressing heads (Figure 5a,b; S1e) reflecting involvement of different metabolic pathways in each case. As for both AD and PD, age is the strongest contributing factor; we propose a two‐hit model to explain the mechanism underlying neurodegenerative diseases where the interaction between the aging induced‐ and Syn/Tau‐ induced reprogrammed metabolic pathways contributes to the pathogenic and clinical symptoms observed in human patients. The “two‐hit” hypothesis was initially proposed by Alfred Knudson in 1971 in the context of tumor suppressor genes explaining the retinoblastoma development by the appearance of two events affecting the tumor suppressor gene RB1 (Knudson Jr., 1971). A similar “two‐hit” hypothesis may be proposed to explain the old‐age onset of neurodegenerative diseases and its possible connection to altered metabolism. Metabolic reprogramming driven by normal aging or by Syn/Tau expression alone causes only mild neurological defects. However, the combination of two hits: aging and Syn/Tau‐ induced metabolic alterations strongly accelerate neuronal degeneration that results in the clinical manifestations. Importantly, targeting of the most affected metabolic pathways might offer an adjunct approach for the treatment of patients with neurodegenerative diseases where simultaneous targeting is likely to be more potent.

### 3.4 Limitation of the study

Because of technical limitations we performed metabolomics on female flies only.

## 4 MATERIALS AND METHODS

### 4.1 Experimental design

*Drosophila melanogaster* reared under controlled laboratory conditions were used to perform targeted metabolomics at different ages (1, 4, and 7 weeks) and at 1 week in case of AD‐, PD, and FTDP‐ models. The resulting data was analyzed and compared to bring out patterns and differences across the data sets. MSEA was used to identify specific metabolic pathways whose metabolites were enriched in each data set. Two approaches were used for the purpose of validation; first was computational data mining of preexisting resources to support our experimental findings and second was genetic manipulations in AD‐ and FTDP‐fly models to confirm modulation of phenotypes upon RNAi induced knockdown of selected genes.

### 4.2 *Drosophila* stocks and maintenance

All flies (*Drosophila melanogaster*) were fed a standard cornmeal media containing yeast, dextrose, sucrose, and molasses and were reared at 25°C in incubators with 12 h light–dark cycle. For all experiments closest genetic control flies were used and were treated the same way as the experimental flies. *Oregon R strain* was used as wildtype flies. For modelling PD, flies expressing human α‐Synuclein under the control of synaptobrevin‐GAL4 were used. For modelling AD and FTDP, flies expressing human wildtype Tau and mutant tau (V337M) respectively under Elav‐GAL4 driver were used. For the eye models of neurodegeneration all the genetic constructs were driven by GMR‐GAL4. All the RNAi lines were procured from the Bloomington *Drosophila* stock center (BDSC). The lines used in the study include BDSC # 43296 (Prat), 44106 (rosy), 51492 (Prat2), and 62216 (GLS). The list of fly stocks used in the study are mentioned in detail in Table S1.

### 4.3 Acquisition of eye pictures and REP quantification

All the flies were aged for 5 days followed by snap‐freezing on dry ice. For each genotype, the compound eye from five to six individual flies were imaged using Nikon SMZ1270 microscope at different focal lengths and were combined later using the freely available CombineZP software. For each genotype, five male and five female flies were used for taking eye pictures.

For quantification, only five flies were used irrespective of sex. For flies expressing Tau^WT^, REP was quantified by scoring eye pictures for the presence or absence of four features namely (i) defect in eye curvature or shape, (ii) presence of fused ommatidia, (iii) presence of irregular sized ommatidia and (iv) loss of ommatidial array. For flies expressing Tau^V337M^, similar scoring was done based on features (i), (ii), (iii) and presence of blebs on the eye surface. Lower scores reflect rescue of the phenotype. Flies expressing Tau^WT^ or Tau^V337M^ had consistent score of 4 demonstrating presence of all four defects in the eye morphology.

### 4.4 Immunoblotting

For immunoblot analysis, 20 fly heads were lysed in RIPA lysis buffer (Cell Signaling) with added phosphatase and protease inhibitors (Roche), using the bullet blender tissue homogenizer. The lysates were resolved on mini‐Protean TGX gel (Biorad) by electrophoresis followed by transfer on PVDF membrane (Immobilon‐P;Millipore), blocked in Tris‐buffered saline Tween‐20 buffer (Cell Signaling Technology) containing 2.5% dry milk and probed with the mentioned antibodies diluted in this buffer. For detecting tau, 5A6 antibody (DHSB) was used at 1:1000 dilution and for synuclein, H3C was used at 1:1000 dilution. For loading control, antibody against β‐Actin (13E5, Cell Signaling technology) and tubulin (T5168, Sigma Aldrich) was used at 1:4000 dilution.

### 4.5 Metabolomics and statistical methods

For metabolomics analysis, flies were collected within 24 h post‐eclosion, sorted by sex under light CO_2_ anesthesia, and reared at standard density (20–25 flies per vial) on fly food at 25°C and 60% humidity with 12 h On/Off light cycle. Flies were transferred to fresh vials every 2–3 days. Ten flies or 35 heads per sample (4–5 biological replicates) were collected under light CO_2_ anesthesia, weighed, and rapidly snap frozen in liquid nitrogen. The intracellular metabolites were extracted using 1.6 mL of cold (−80°C) 80% (v/v) aqueous methanol. The tissue was homogenized using 0.5 mm zirconium beads, and insoluble material in the lysates was centrifuged at 5000*g* for 5 min. The resulting supernatant was evaporated using a speed vac. Samples were resuspended using 20 μL of HPLC grade water for mass spectrometry. 5 μL of sample was injected and analyzed using a 6500 QTRAP triple quadrupole mass spectrometer (AB/SCIEX) coupled to a Prominence UFLC HPLC system (Shimadzu) via selected reaction monitoring (SRM) of a total of 302 endogenous water‐soluble metabolites for steady‐state analyses of the samples. Some metabolites were targeted in both the positive and negative ion mode for a total of 302 SRM transitions using positive/negative polarity switching. ESI voltage was +4900 V in the positive ion mode and − 4500 V in the negative ion mode. The dwell time was 3 ms per SRM transition, and the total cycle time was 1.35 s. Approximately 10–14 data points were acquired per detected metabolite. Samples were delivered to the mass spectrometer via normal phase chromatography using a 4.6 mm i.d × 10 cm Amide Xbridge HILIC column (Waters Corp.) at 350 μL/min. Gradients were run starting from 85% buffer B (HPLC grade acetonitrile) to 42% B from 0 to 5 min; 42% B to 0% B from 5 to 16 min; 0% B was held from 16 to 24 min; 0% B to 85% B from 24 to 25 min; 85% B was held for 7 min to reequilibrate the column. Buffer A was comprised of 20 mM ammonium hydroxide/20 mM ammonium acetate (pH = 9.0) in 95:5 water: acetonitrile. Peak areas from the total ion current for each metabolite SRM transition were integrated using MultiQuant v3.0 software (AB/SCIEX).

The resulting raw data from the MultiQuant software was uploaded in Metaboanalyst v 4.0 (www.MetaboAnalyst.ca), and subsequent data processing and analyses were performed using this tool. Metabolites that were not detected in 40% of the samples were excluded from the analysis. Data were normalized to the median (per sample) and processed through log transformation. A heat map with hierarchical clustering was generated using Pearson correlations and Ward's method. MSEA was performed using Metaboanalyst, which uses the KEGG pathway database (http://www.genome.jp/kegg/pathway.html). Metabolite sets containing at least 5 compounds were employed in the analysis. MSEA calculates hypergeometrics test scores based on cumulative binominal distribution.

### 4.6 Analysis of the enriched metabolic pathways in the human AD, PD, and AD/PD samples

From the metabolomic analysis of the three different models in *Drosophila*, we identified several significantly affected metabolic pathways using the KEGG database. We tested whether the human orthologs of the *Drosophila* enzymes that belong to the affected metabolic pathways were also significantly affected in human brains. Briefly, we retrieved the KEGG pathways and the associated enzymes, and tested whether their levels are significantly different in human brains by using a publicly available human dataset that has the data for AD, PD, and AD+PD proteomes. We generated the heatmaps using the “ComplexHeatmap” package (Gu et al., 2016). We also tested whether the number of enzymes, which are significantly changed for the particular pathway, is higher than simply by chance.

## AUTHOR CONTRIBUTIONS

*Conceptualization*: PA. *Methodology*: PA, YS. *Investigation*: PA. *Visualization*: PA, YS. *Supervision*: PA. *Writing—original draft*: PA, YS. *Writing—review & editing*: PA, YS.

## FUNDING INFORMATION

This work was supported by NIGMS R35 GM146869 (P.A.), NIA R00 AG057792 (P.A.), NIA R03 AG075651 (P.A.), NIA P30 AG024827 pilot grant (P.A.), and NIH P01CA120964 (J.M.A.). This research was also supported in part by the University of Pittsburgh Center for Research Computing through the HTC cluster, which is supported by the NIH award S10OD028483.

## CONFLICT OF INTEREST STATEMENT

The authors declare that they have no competing interests.

## Supporting information


Figure S1.

Table S1.

Table S2.

Table S3.

## Data Availability

All data are available in the main text or the supplementary materials.
