# Supplementary figures and images for: Unique tau‐ and synuclein‐dependent metabolic reprogramming in neurons distinct from normal aging
Source: Aging Cell. 2024 Aug 13;23(11):e14277. doi: 10.1111/acel.14277 (PMC11561663; doi:10.1111/acel.14277)

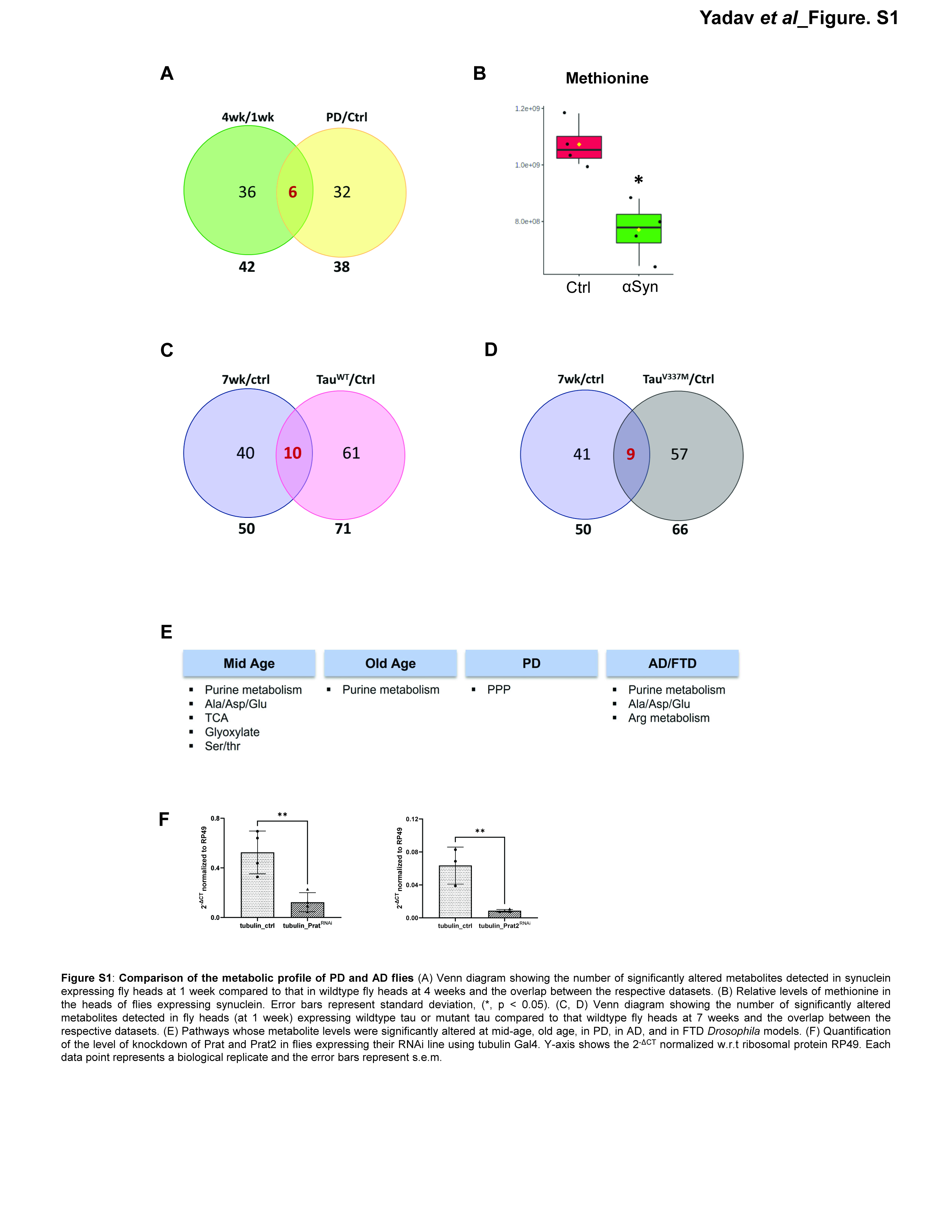

Supplement: Supplementary file 1 — Figure S1. [file ACEL-23-e14277-s001.tif]
